# Development of a Mobile Health Application for HIV Prevention Among At-Risk Populations in Urban Settings in East Africa: A Participatory Design Approach

**DOI:** 10.2196/23204

**Published:** 2021-10-07

**Authors:** Wilhellmuss Mauka, Christopher Mbotwa, Kåre Moen, Hanne Ochieng Lichtwarck, Inga Haaland, Method Kazaura, Germana H Leyna, Melkizedeck T Leshabari, Elia J Mmbaga

**Affiliations:** 1 Department of Behavioural Science Muhimbili University of Health and Allied Sciences Dar es Salaam United Republic of Tanzania; 2 Ministry of Health, Community Development, Gender, Elderly and Children Dodoma United Republic of Tanzania; 3 Department of Epidemiology and Biostatistics Muhimbili University of Health and Allied Sciences Dar es Salaam United Republic of Tanzania; 4 Mbeya College of Health and Allied Sciences University of Dar es Salaam Mbeya United Republic of Tanzania; 5 Department of Community Medicine and Global Health Faculty of Medicine, Institute of Health and Society University of Oslo Oslo Norway; 6 Tanzania Food and Nutrition Centre Dar es Salaam United Republic of Tanzania

**Keywords:** mHealth application, participatory design, HIV, pre-exposure prophylaxis, Africa, female sex workers, sex and gender minorities

## Abstract

**Background:**

There is limited evidence in Africa on the design and development of mobile health (mHealth) applications to guide best practices and ensure effectiveness. A pragmatic trial for HIV pre-exposure prophylaxis roll-out among key populations in Tanzania is needed.

**Objective:**

We present the results of the development of a mobile app (Jichunge) intended to promote adherence to pre-exposure prophylaxis (PrEP) among men who have sex with men (MSM) and female sex workers (FSW) in Tanzania.

**Methods:**

A participatory design approach was employed and guided by the information system research framework. MSM and FSW were the target populations. A total of 15 MSM and 15 FSW were engaged in the relevance and design cycles, while the piloting phase included 10 MSM and 20 FSW.

**Results:**

The relevance cycle enabled the description of the existing problem, provided the compatible app features for the target population, and identified the need to develop an mHealth app that provides health services in a stigmatizing and discriminating environment. User involvement in the app’s design and evaluation provided an opportunity to incorporate social, cultural, and community-specific features that ensured usability. In addition, the participants suggested valuable information to inform the app, text message services, medication registration, and chat platform designs.

**Conclusions:**

The participatory design approach in the development of mHealth apps is useful in identifying and validating population-specific functional features, improve usability, and ensuring future health impacts. Through this participatory process, the Jichunge app took end-user needs, perspectives, and experiences into account, eliciting enthusiasm regarding its potential role in supporting pre-exposure prophylaxis adherence for HIV and related behavioral change promotion.

**Trial Registration:**

International Clinical Trials Registry Platform PACTR202003823226570; https://trialsearch.who.int/Trial2.aspx?TrialID=PACTR202003823226570

## Introduction

As part of a pragmatic trial in Tanzania, this paper describes the process of developing a mobile health (mHealth) application to improve adherence to pre-exposure prophylaxis (PrEP) against HIV among men who have sex with men (MSM) and female sex workers (FSW).

Over the last decade, there has been a steady increase in global mobile phone ownership, including sub-Saharan Africa, which has led to remarkable growth in mobile device use in curative and preventive health care service delivery [1,2]. Short text–based messaging was the mainstay of mobile health communication in the past. However, smartphone applications with increased functionality have become more commonplace in recent years. They have been used to prevent disease and promote health, including sexual and reproductive health [3-7]. mHealth apps have been reported to be useful in disease self-management, customized to clients’ and local needs, and providing on-demand interventions as well as communitywide health promotion [8,9].

Among other things, mHealth apps have been used to improve drug adherence in HIV care by reminding clients to take a particular medication, providing health education, and counseling and services [10-12].

A systematic review on mHealth apps in low-income and middle-income countries has reported increased use of various apps focusing on patient care, supporting the health system, and general population health [13,14].

In 2015, the World Health Organization recommended PrEP for HIV prevention among at-risk populations [15]. Like other parts of the world, poor adherence to medication among the high-risk population has been a significant challenge to implementing PrEP intervention in Africa [16-18]. In 2018, Tanzania initiated plans for a PrEP roll-out, but suboptimal adherence due to stigma, stock shortages, forgetfulness in taking daily pills, or attending clinics for medication refills pose a challenge to its effectiveness [19,20].

Since more than half of the mobile subscribers in Tanzania have access to the internet [21], and social network usage is common [22], mHealth apps offer an opportunity to promote adherence to PrEP in the country.

The reported increase in mobile apps usage for health calls for the documentation of best practices in the development process of these interventions. Such evidence can guide future development and increase the effectiveness of mHealth apps in promoting health. Various development approaches have been evaluated in Asia and the United States, several of which have underlined the critical importance of end-user involvement in the design and development process [23-25]. End-user involvement is paramount when developing mHealth apps targeting populations that face a high level of stigma and discrimination, such as MSM and FSW. While mobile apps have increasingly come into use among stigmatized and criminalized people in sub-Saharan Africa, there is limited data on the development process to inform best practices in the region and among these populations [26]. This paper aims to present the development process of an mHealth app (Jichunge app) for PrEP adherence among MSM and FSW in Tanzania.

## Methods

### Design

**Figure 1:**
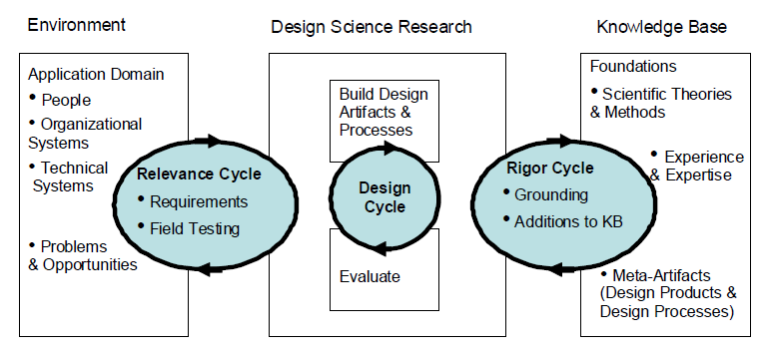
The information system research framework. KB: Knowledge Base.

A participatory design approach guided by a framework reminiscent of the information system research framework (ISR) was employed in the Jichunge app development [27]. ISR emanates from design science and system engineering commonly used to develop complex information systems [28]. The framework urges end-users' greater inclusion and active participation in designing and evaluating application systems [23,29]. The ISR framework consists of three overarching user participatory design cycles. The relevance cycle determines end-user requirements; the design cycle involves prototype development and evaluation; the rigor cycle focuses on assessing underpinning theories (Figure 1). This paper focuses on the relevance and design cycles that are critical in end-product acceptability and usability. Therefore, user involvement was paramount in these undertakings. We supported end-user involvement in the conceptualization, design, and testing by considering their views, perspectives, comments, and suggestions at different levels of the participatory continuum [30].

### Population

MSM (men who regularly or occasionally have sex with men) and FSW (women who exchange sex for money or goods) living in Dar es Salaam, Tanzania, participated in this study as members of the target population or end-users. Eligibility involved being at least 18 years and a resident of Dar es Salaam, the largest metropolitan city in Tanzania. The resident was defined as having an address and having lived in the city for the past 6 months preceding the study.

Additionally, at each stage of development, the app was reviewed by a team of experts in HIV behavior and research in key populations. The prototype evaluators involved a multidisciplinary team consisting of PhD students, medical researchers, epidemiologists, biostatisticians, public health specialists, and clinical research coordinators.

### Participant Sampling and Recruitment

Purposive sampling was used to enroll members of the target population (participants) into the study. Based on the investigators' previous knowledge of working with the target populations, participants were recruited based on their potential to provide rich information on the issues related to PrEP use, challenges, and opportunities. Moreover, different strata of the population by age and geographical location in the city, mobile phone ownership, leadership position within target population grassroots organizations, and economic structures were critical in providing diverse views on PrEP and mobile phone use to address health challenges among the target population.

### Development Process

#### Relevance Cycle

The study gathered and synthesized information related to the Jichunge app's relevance to the target populations through the relevance cycle step. Firstly, the investigators consulted members of the target populations for problem definition and justification of the app's relevance, including need and potential challenges. End-users’ consultation aimed to explore their views on adherence to PrEP and whether a mobile app would be relevant in addressing those challenges. Secondly, using investigators' experience working with these population subgroups [31-34], a detailed and appropriate literature review was conducted to validate the challenges in PrEP adherence raised and explore the proposed Jichunge app's needs to address PrEP adherence among the members of the target populations. Furthermore, researchers reviewed literature on mobile phone access and usage involving the target population, using available social media platforms to inform the design and assess future application challenges and opportunities.

#### Design Cycle

##### Creating an App Prototype

In creating the app prototype, consultation with target populations led to the consideration of developing an app that accommodates both iOS and Android smartphone operating systems available in Tanzania. Due to the potential for limited mobile data among the key population, users recommended developing an application that allows access to app features such as notification messages (reminding them to take their medication) even in the absence of mobile data. Similarly, the app should enable the client to register daily pills taken for drug adherence, monitoring, and virtualizing the pill calendar even when the user lacks mobile data. An app's development with these innovations was led by a company based in Norway with vast software development experiences [35].

##### Stage I of Testing the Prototype and Modifications: Prototype Preliminary Reviews

The initial prototype was installed into investigators’ mobile phones as a demonstration version with the essential functions. Next, the investigators reviewed the various app functionalities and evaluated their relevance per planned use among the intended study populations.

##### Stage II of Testing the Prototype and Modifications: Target Populations Design Inputs

**Figure 2:**
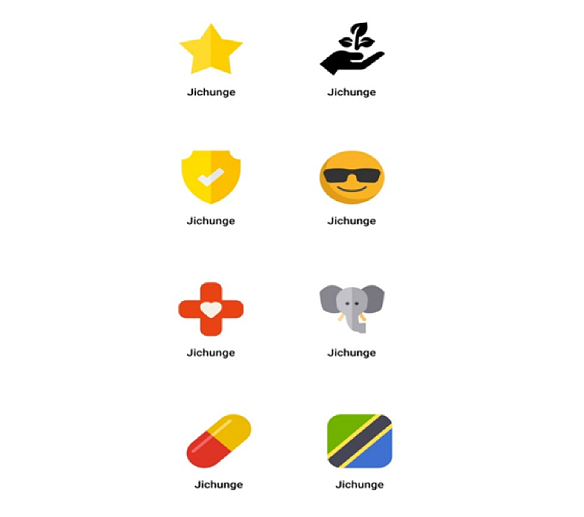
Proposed and designed logos.

Various external outlooks and logos were designed (Figure 2) and shared with 15 members from each end-user group (MSM and FSW) in informal, exploratory individual and small group discussions. The participants were FSW and MSM with a mean age of 26 years (IQR 20-48), the majority (18/30; 60%) had completed primary school, and 6.6% (2/30) had a college diploma. In addition, most of the participants (21/30; 70%) were self-employed.

**Figure 3:**
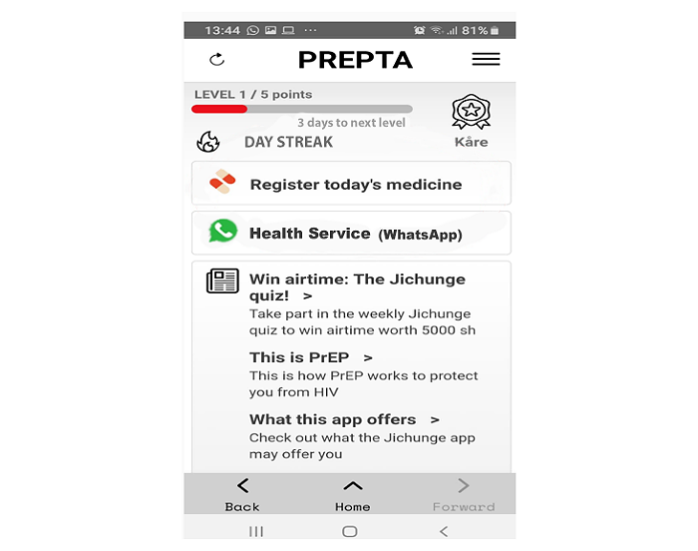
Previous outlook and name of the app. PrEP: pre-exposure prophylaxis.

User involvement at this design stage aimed to receive user perspectives, ensure their needs are met, and explore anticipated challenges to inform the app design. End-users’ discussion topics in small groups included the app name, layout, and functions. Figure 3 illustrates the sample outlooks and app name as proposed by end-users. All suggestions provided by the end-users were submitted to the software developer for app modification. The intended outcome was to develop an outlook and application name, which is original, user-friendly, and stigma and discrimination-free.

#### Rigor Cycle

##### Piloting of the Jichunge App

The pilot was carried out in Dar es Salaam, where the app will be rolled out and evaluated. FSW and MSM end-user populations were recruited through their peers to participate in the app's piloting phase.

We conducted 2 focus group discussions (FGD) before and after using the app to explore participants’ perceptions, experiences, and challenges associated with using the Jichunge app. The FGDs involved 20 members of the HIV at-risk populations (10 FSW and 10 MSM). The participants' median age was 27 years (IQR 21-44), the majority (12/20; 70%) had completed primary school, and 65% (13/20) were self-employed. Two PhD students led the FGDs, one as a facilitator and the other as a notetaker.

A demonstration of the app’s functionalities and operations was performed for the users, and they were later invited to discuss those functionalities, including expectations regarding the app use. Each of the FGDs took an average of one hour.

**Figure 4:**
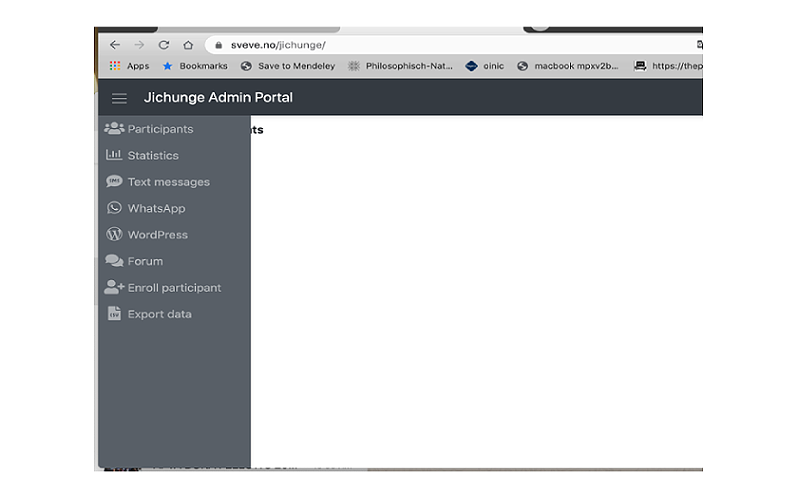
Jichunge app web-based admin portal.

Following the FGDs, the Jichunge app was provided to the participants. The app is only available by invitation to ensure that only the target populations can access it and maintain confidentiality. Participants were registered in the web-based portal during the installation, using their mobile phone numbers as illustrated in Figure 4. The installation process took 10 to 20 minutes, depending on the phone type and internet connectivity.

After installation, participants were given 2 weeks to use the app, and they had the opportunity to communicate with a doctor, one of the researchers, and a designated peer educator. During this period, they were also able to consult the research team and the software company.

At the end of the second week, participants were invited for the second round of FGDs. Two FGDs, one for MSM and one for FSW, were conducted. Discussions focused on their experiences, perspectives, and challenges regarding using each of the app’s functionalities and features. Suggestions for improvement were also presented to the software developer for improvement.

##### Usability Measures

The usability was quantitatively measured through user statistics of various app features recorded in the app web-based server. Data were extracted from the web-based server into Microsoft Excel, then entered into Stata (version 13.1; StataCorp) for analysis. The mean number of visits to each of the 9 app features per day was estimated and used to measure and monitor the app's usability.

### Data Analysis

All qualitative data from FGDs and informal exploratory discussions were done in Swahili, the language spoken by all Tanzanians. Data from FGDs were recorded and transcribed verbatim. Field notes were taken during the exploratory individual or small group discussions. Data were later translated to the English language before conducting a content analysis. We applied an open coding approach during the initial data collection stage, where emerging subthemes were identified. Various emerging and related subthemes were subsumed into broader themes relevant to the app's design, functions, and usability. These themes were later summarized and discussed by at least 2 researchers. Relevant quotes for each of the themes were summarized and used in the manuscript. Descriptive analysis was done for quantitative data. Proportions were calculated for categorical variables, and mean or median were estimated for continuous variables as deemed appropriate. The study was approved by the National Health Research Ethics committee, Tanzania (NIMR/HQ/R.8a/Vol.IX/3454).

## Results

### Relevance Cycle

The results are presented based on the ISR framework cycles adopted in the Jichunge app development process. End-users’ consultation revealed that the high demand for developing and implementing innovative approaches that can assist key populations in health-related matters, including improving uptake and adherence to PrEP. Members of the target population enthusiastically received the idea and expressed their willingness to support such development. Users indicated the need to have a culturally and legally sensitive application that would provide anonymity and safety and yet offer a high level of desired functions to accommodate such anonymity. Following users’ suggestions, the investigators proposed indistinct terms to label the target population groups in the app. The literature review indicated limited access to health services among HIV at-risk populations, including FSW and MSM in the African setting [33]. Access to health information and services among these populations in mainstream public health facilities is associated with many difficulties due to stigma and discrimination. Poor adherence to PrEP among at-risk populations was also reported and threatened the effectiveness of this preventive intervention [26,34,36].

### Jichunge App Main Functionalities

**Figure 5:**
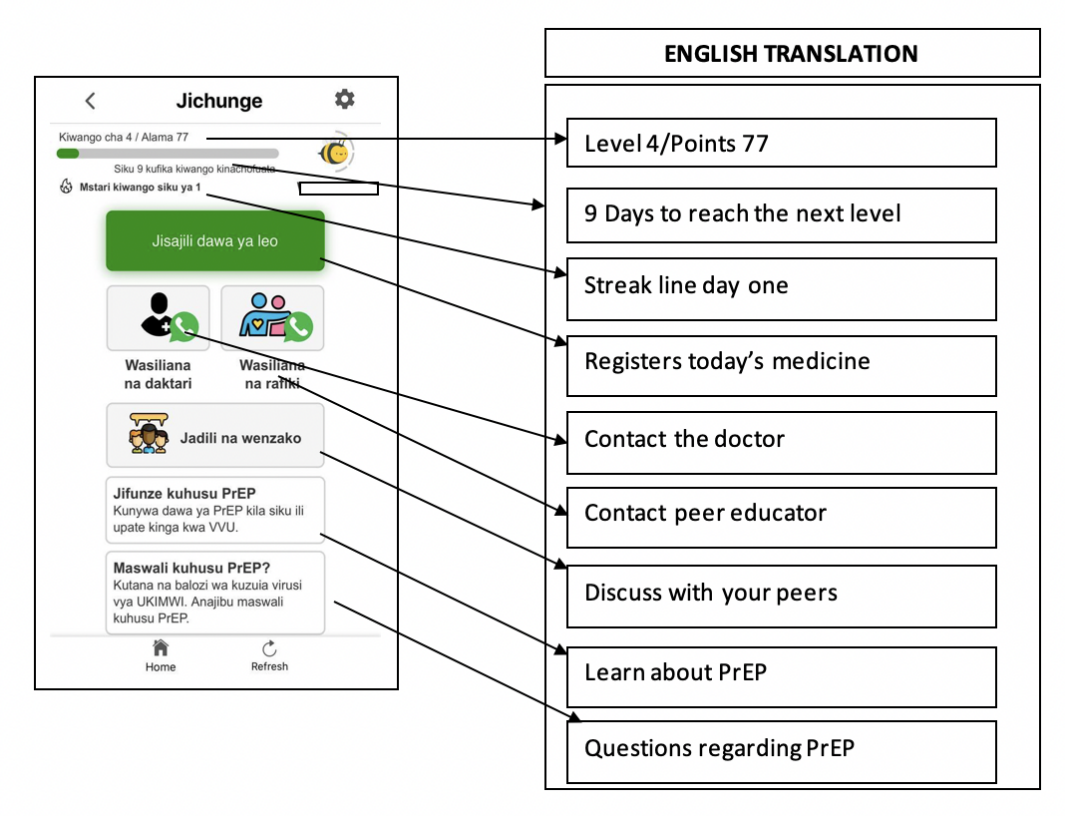
Final outlook and functionalities of Jichunge app. PrEP: pre-exposure prophylaxis.

The final prototype of the Jichunge app has 10 various functionalities or features, as presented in Textbox 1. The outlook of the app features to the users is displayed in Figure 5. The Jichunge app can provide information on the participant’s level of adherence to daily PrEP pills, allow gamification and winning of prizes, register pills taken, allow setting up of timed reminder messages, send a customized reminder message to the user, provide a platform for real-time communication between the user with a peer educator or a health care worker, provide health education, and allow participation in various quizzes.

**A summary of the Jichunge app’s main functionalities. PrEP: pre-exposure prophylaxis.**

**Levels and points:** Shows a person’s level of adherence per month.

**Gamification:** Provides a virtual view of the medication registration progress and displays a winning trophy that automatically enters the user in gamification, allowing them to win various prizes, including mobile airtime, data, and others.

**Drug registration:** The user registers each time they take the medication for records.

**Medication time reminder:** The user can set up a personal time to be reminded to take the medication daily.

**Notification:** It notifies the user to take and register the medication per the medication reminder.

**Communication with peers (WhatsApp feature):** It offers the users an opportunity to communicate in real-time with a trained peer educator about PrEP and other health service-related opportunities and challenges. They are also able to discuss different social issues of relevance to the key population for HIV.

**Communication with a health care provider (WhatsApp):** It offers users an opportunity to communicate in real-time with a health care provider (doctor) on PrEP and other health-related issues.

**Discussion forum (chat room feature):** A platform where users can chat with peers anonymously using autogenerated aliases.

**Educational materials (WordPress feature):** The Jichunge app will contain educational documents and pictorial presentations providing information on learning about PrEP and common questions regarding PrEP. This platform provides users with information on PrEP-related issues.

**Jichunge quiz:** Users can take a quiz and have a chance to win airtime.

### Rigor Cycle

#### Participants’ Reported Experiences Using the Jichunge App

Overall, participants expressed their satisfaction with the Jichunge app and expressed their readiness to continue app use. Some reported having proposed the app’s usefulness to their peers who were not part of the pilot study. Participants praised the reminder messages as being extremely useful. Even if they did not have internet connectivity or airtime to receive the message, one participant stated:

I have never received the message but, since I like to use the phone, once I see the app’s logo, I remember to take medicine.

FSW FGD, participant 1

Access to educational content about PrEP and other HIV-related information was of most interest to the participants. In addition, the app served as a reference and source of information whenever misunderstandings among members of the at-risk population arose.

…I learn and know more about PrEP, you find many things, considering there are things we are asked in the group. If there is a question that has been asked and you don’t know the answer, you go there (into the app), check, and come back to tell your colleagues.

MSM FGD, participant 1

Both MSM and FSW were excited about the app. They reported that the outlook (logo) of the app, its features, and built-in anonymity (members only) make it secure and confidential, protecting their discussions and identities. An FSW from the FGD narrated.

…This is going to keep our secrets because they will not be able to just get it like eh! this WhatsApp. For us, it looks like a baby app, the logo leaf, we are mothers, so this is good and secretive.

FSW FGD; participant 6

Users who provided feedback generally expressed positive attitudes and acceptability of the app design and functions. Suggestions for app improvement included redesigning the discussion forum to match the WhatsApp platform, with which many study participants were acquainted. Users also had some suggestions on the features that should be improved, including the appearance of chat forums on its appearance and showing current online users.

…This discussion forum is not somehow appealing…Once you enter, you should at least know how many are online…Sometimes you don’t see the message of other people.

MSM FGD, participant 3

Moreover, users proposed changes in the gamification score indicators to resemble everyday objects in their environments, such as a ladder, speedometer, or a trophy used in football games. End-users endorsed the app's name, Jichunge*,* a Swahili word meaning “protect yourself.” Population-specific and sensitive language translation from English to Swahili (Tanzania’s national language) was also incorporated in the app.

#### Usability Measures

**Figure 6:**
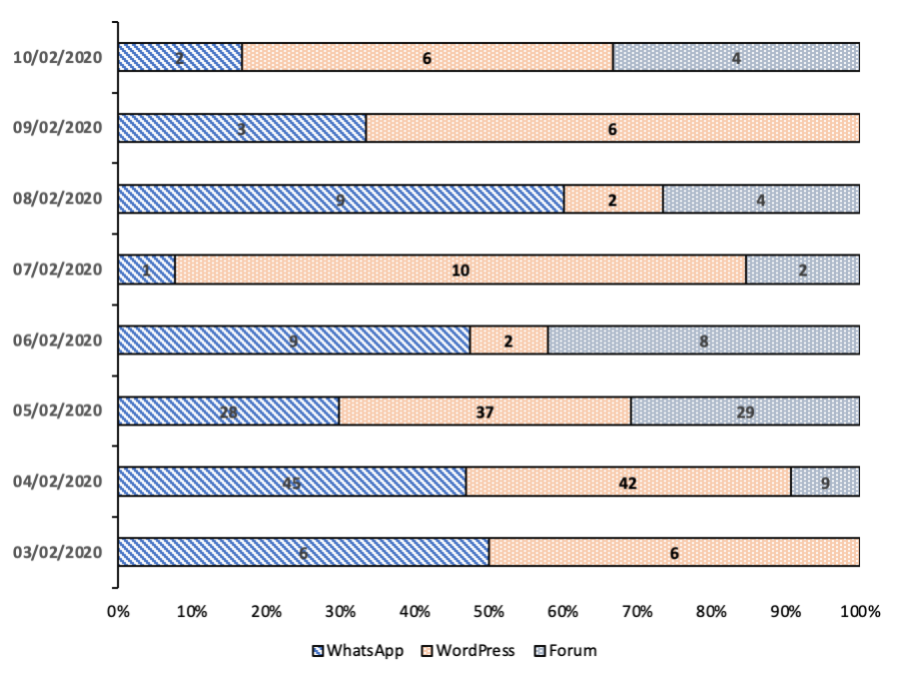
The number of App features used per day 3-10 February 2020 (N=270).

The Jichunge app pilot study recorded 68 pill registrations during the first 7 days, and on average, half of the users (10/19, 52%) registered the medicines taken per day. A third of the users (6/19, 31.6%) had complete registration of their daily pill taking. There were variations in daily pills registrations among the 19 participants, with 6 (31.6%) registering during the first day and 12 (63.2%) on the second day, followed by 8 (42.1%), 11 (57.9%), 10 (52.6%), 11 (7.9%), and 12 (63.2%) during subsequent days (Table 1). The users used, on average, 1 out of 9 app features per day. The features visitation rates were highest for WhatsApp (100/270, 37.0%) and WordPress (102/270, 37.8%). WhatsApp’s platform provided secure communication with a peer educator or a doctor, while WordPress contained educational materials about PrEP (Figure 6).

**Table 1:** Number of pill registrations per day during the first week of February 2020.

Day	Pill registrations per day (N=19), n (%)
1	6 (31.6%)
2	12 (63.2%)
3	8 (42.1%)
4	11 (57.9%)
5	10 (52.6%)
6	11 (57.9%)
7	12 (63.2%)

## Discussion

### Main Findings

This paper presents results from a participatory design approach in developing the Jichunge mobile app to improve adherence to PrEP among MSM and FSW in Tanzania. The app includes various functionalities that encourage not only adherence to PrEP but also HIV preventive behaviors. The final version of the application offers 10 unique features, as presented in Textbox 1. The development process, focusing on the relevance and design cycles of the ISR framework, ensured optimal end-user participation in the design and evaluation. MSM and FSW are stigmatized and discriminated against in the sub-Saharan region and Africa as a whole. Their behaviors are deemed illegal, limiting access to population-specific health services [26,34,36]. Therefore, innovative approaches are needed to facilitate access and adherence to health-related services such as PrEP for HIV prevention. Studies have indicated that mobile applications developed with adequate consideration of intended users’ needs and preferences are more likely to be utilized [37]. Hou et al [38] also stressed the importance of user-centered involvement in developing relevant mHealth apps and ensure usability. The user participatory approach provides an opportunity to validate proposed app features and identify relevant functional features for inclusion in the final design to ensure effectiveness [27,39]. In the Jichunge app, users provided critical inputs on the design, including logo, app name, and features. The participants suggested several modifications, such as text delivery format, gamification, and improvements in the discussion forum. A user-accepted outlook (logo) may go a long way in impacting service use by merely visualizing their phone’s logo. An appealing logo stresses the importance of a user-friendly app’s appearance in eHealth and mHealth technologies [3].

As expected, the high usability of the application was observed on the daily medicine registration feature. Given that this was the first time these populations in Tanzania used an mHealth application, the results are promising. Moreover, they indicate the potential for future optimal usability. Therefore, an innovative interactive mHealth application may go a long way in strengthening access to health information and access to health services among these populations.

The discussion forum suggested an improvement to include virtualization of other online members, which was considered in the final design to provide a sense of community engagement [40].

Among the lessons learned during the introduction and onboarding of participants into the app was ensuring wireless internet connectivity, which reduces participants' internet costs. Moreover, to ensure that participants have a better opportunity to learn more about the app, promotional posters, app functionality fliers, and demonstration videos should be considered alongside verbal descriptions [41,42]. Finally, in a limited resource setting, planning for mobile application intervention may be hindered by outdated versions of mobile phones, which may not be compatible with most modern applications.

### Limitations

The results of this study should be interpreted in light of the following limitations. Firstly, the usability measurement was done through the number of visits to each feature by the user. However, we acknowledge that visit statistics for some features, such as educational resources, may not indicate detailed (quality) reading. It would be more informative to have measures such as design layout, comprehension of learning, and application performance.

Secondly, at both the app design and pilot stage, we had exploratory interviews with only 30 participants and conducted 2 focus group discussions involving 20 participants. Although these participants' sociodemographic characteristics did not differ from that of the user population in the city, we acknowledge that the participants may not represent the views and perspectives of the entire population of FSW and MSM. We used strategic peer-led recruitment (respondent-driven sampling) as the recommended recruitment method in stigmatized, criminalized, and hard-to-reach populations.

Thirdly, the app's reporting of drug use may be affected by a desirability bias, where people may decide to register treatment administration without taking the tablet. Similarly, there could be people who have used the tablet but did not register. As in many desirability-prone studies, confirmatory measures such as pill counts and biological markers are recommended and planned in our future trial using the Jichunge app.

### Conclusions

The participatory design approach in mHealth app development helps assess and cooperate end-users’ experiences and perspectives and identify potential challenges to guide future app usability. The Jichunge app accounted for the sociocultural and legal context of the target populations eliciting enthusiasm with the potential to impact adherence to PrEP for HIV prevention and general risk-free behavioral promotion.

## Abbreviations

EDCTP2: European and Developing Countries Clinical Trials Partnership
FGD: focus group discussion
FSW: female sex workers
GLOBVAC: global health and vaccination
ISR: information system research framework
mHealth: mobile health
MSM: men who have sex with men
PrEP: pre-exposure prophylaxis
